# Room temperature, ppb-level NO_2_ gas sensing of multiple-networked ZnSe nanowire sensors under UV illumination

**DOI:** 10.3762/bjnano.5.194

**Published:** 2014-10-22

**Authors:** Sunghoon Park, Soohyun Kim, Wan In Lee, Kyoung-Kook Kim, Chongmu Lee

**Affiliations:** 1 Department of Materials Science and Engineering, Inha University, 253 Yonghyun-dong, Nam-gu, Incheon 402-751, Republic of Korea; 2 Department of Chemistry, Inha University, 253 Yonghyun-dong, Nam-gu, Incheon 402-751, Republic of Korea; 3 Department of Nano-Optical Engineering, Korea Polytechnic University, 2121 Jeongwang-dong, Shiheung city, Gyeonggi-do, 429-793, Republic of Korea

**Keywords:** gas sensors, NO_2_, UV, ZnSe nanowires

## Abstract

Reports of the gas sensing properties of ZnSe are few, presumably because of the decomposition and oxidation of ZnSe at high temperatures. In this study, ZnSe nanowires were synthesized by the thermal evaporation of ZnSe powders and the sensing performance of multiple-networked ZnSe nanowire sensors toward NO_2_ gas was examined. The results showed that ZnSe might be a promising gas sensor material if it is used at room temperature. The response of the ZnSe nanowires to 50 ppb–5 ppm NO_2_ at room temperature under dark and UV illumination conditions were 101–102% and 113–234%, respectively. The responses of the ZnSe nanowires to 5 ppm NO_2_ increased from 102 to 234% with increasing UV illumination intensity from 0 to 1.2 mW/cm^2^. The response of the ZnSe nanowires was stronger than or comparable to that of typical metal oxide semiconductors reported in the literature, which require higher NO_2_ concentrations and operate at higher temperatures. The origin of the enhanced response of the ZnSe nanowires towards NO_2_ under UV illumination is also discussed.

## Introduction

ZnSe has been widely used in fabricating short-wave optoelectronic devices [1] including blue–green laser diodes [2], tunable mid-IR laser diodes for remote sensing [3], white-light LEDs [4], continuous wave ZnSe-based laser diodes [5] and UV photodetectors [6]. On the other hand, there are almost no reports on the gas sensing properties of ZnSe. This might be due to the decomposition and oxidation of ZnSe at temperatures above 200 °C [7] and a lack of good sensing performance at room temperature.

In recent years, one-dimensionally (1D) nanostructured, metal oxide semiconductor sensors have been studied extensively because of the associated higher sensitivity due to the high surface-to-volume ratios as compared to thin film gas sensors [8–13]. Most metal oxides exhibit some sensitivity to many gases at high temperatures because gas sensitivity tends to increase with increasing temperature. On the other hand, the development of highly sensitive and selective sensors at room temperature is still a challenge. Several techniques including the doping [12,14–15] or surface functionalization [16–18] of metal catalysts, core–shell structure formation [19–21] and UV irradiation [22–24] have been developed to improve the sensing performance, detection limit and selectivity of 1D nanostructure sensors at room temperature. Among these techniques, the UV illumination method was used in the present study to enhance the sensing performance of ZnSe, 1D nanostructure-based sensors at room temperature. In this study, multiple-networked ZnSe nanowire sensors were fabricated and examined for their room-temperature, NO_2_-gas sensing properties under UV illumination. Unlike individual 1D nanostructure sensors, multiple-networked 1D-nanostructured sensors have the benefits of low sensor fabrication cost (because there is no need for precise techniques to connect the nanostructures), as well as outstanding sensing performance.

## Results and Discussion

### Analysis of the structure of ZnSe nanowires

**Figure 1:**
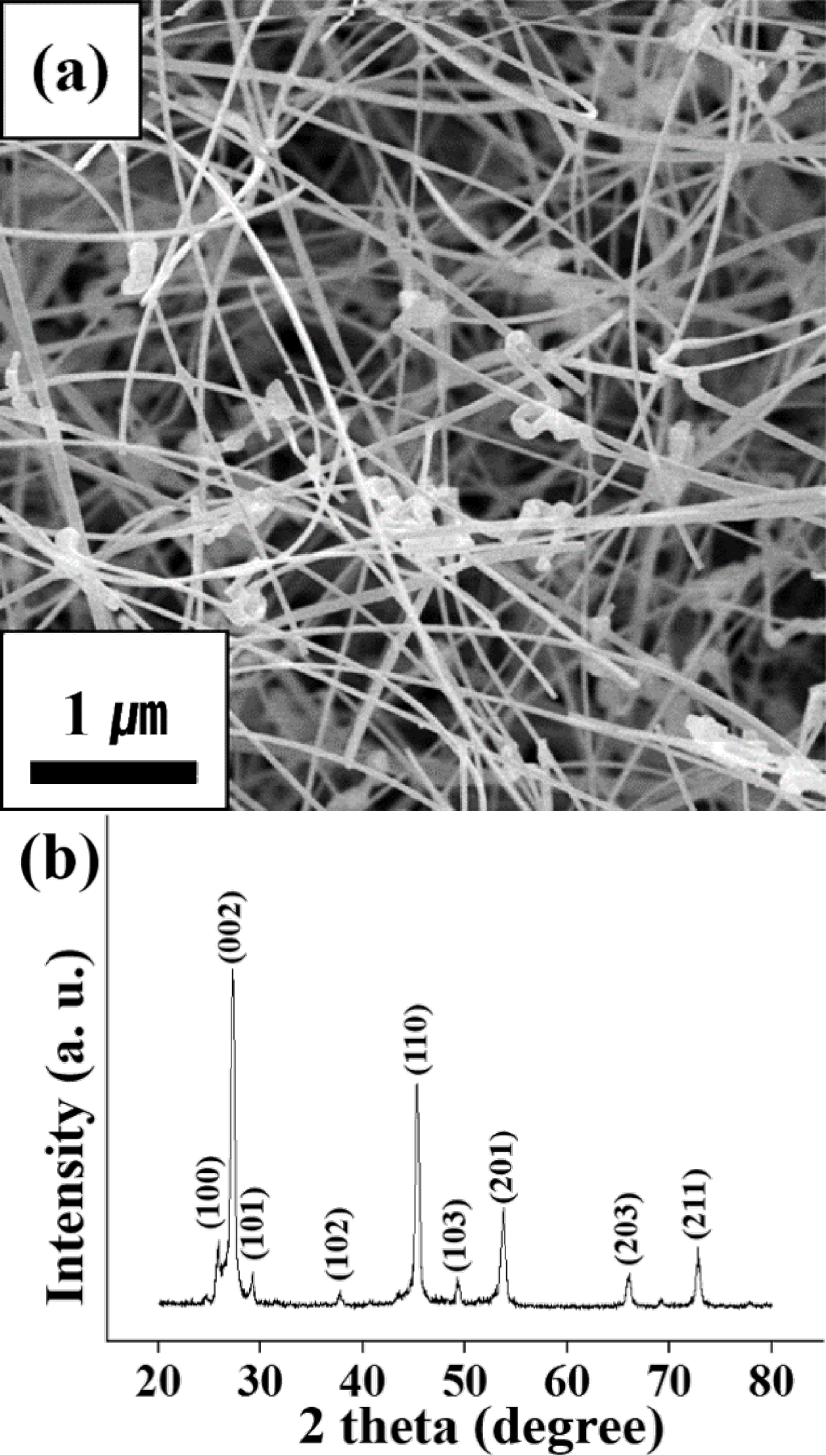
(a) SEM image of ZnSe nanowires. (b) XRD pattern of the ZnSe nanowires.

Figure 1a shows a SEM image of the ZnSe, 1D nanostructures. The 1D nanostructures exhibited a wire- or fiber-like morphology with widths ranging from 30 to 100 nm and lengths ranging up to ≈300 μm. Figure 1b shows the corresponding XRD pattern of the ZnSe nanowires. The XRD pattern of the ZnSe nanowires showed six sharp reflection peaks assigned to wurtzite-structured ZnSe with lattice constants of *a* = 0.3996 nm and *c* = 0.6626 nm (JCPDS No. 89-2940), suggesting that the nanowires were crystalline.

**Figure 2:**
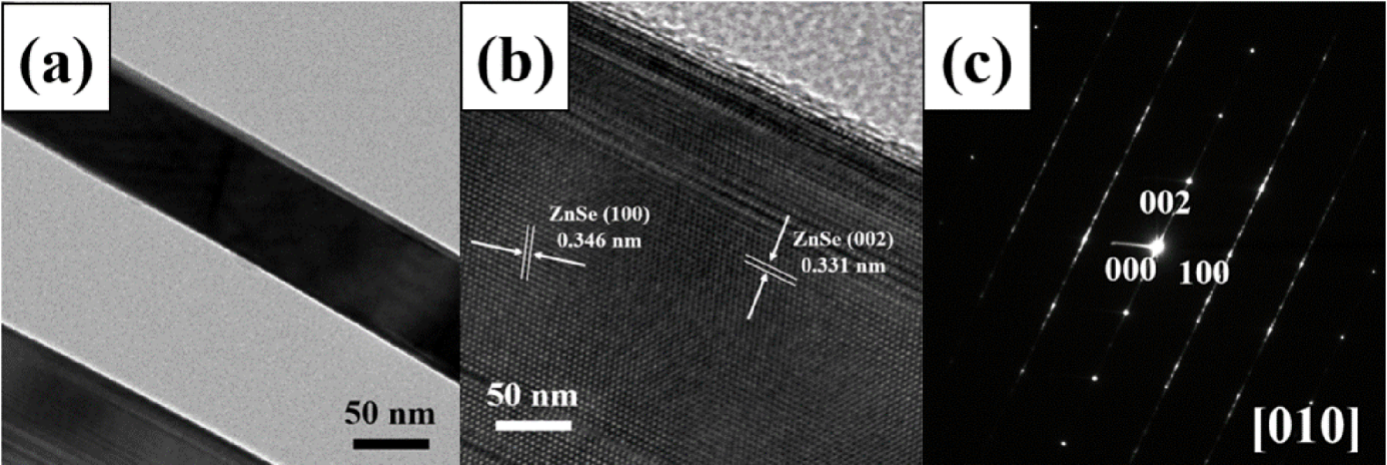
(a) Low-magnification TEM image of a typical ZnSe nanowire, (b) high-resolution TEM image of a typical ZnSe nanowire and (c) corresponding SAED pattern.

The low-magnification TEM image in Figure 2a revealed a typical ZnSe nanowire with an extremely uniform diameter of approximately 80 nm. The HRTEM image in Figure 2b confirmed that the core region of the nanowire was perfectly crystalline, whereas the edge region showed twinning along the axis of the nanowire. Fringes with spacings of 0.346 and 0.331 nm corresponding to the interplanar distances of the {100} and {002} lattice planes, respectively, were clearly observed in the core region. The corresponding selected area in the electron diffraction pattern (Figure 2c) exhibited two types of reflection spots assigned to wurtzite-structured ZnSe: a round reflection from the core region and an elongated reflection from the edge region.

The corresponding selected area electron diffraction pattern (Figure 2c) exhibited two types of reflection spots assigned to wurtzite-structured ZnSe: round one from the core region and elongated one from the edge region.

### Performance of nanowire gas sensors

**Figure 3:**
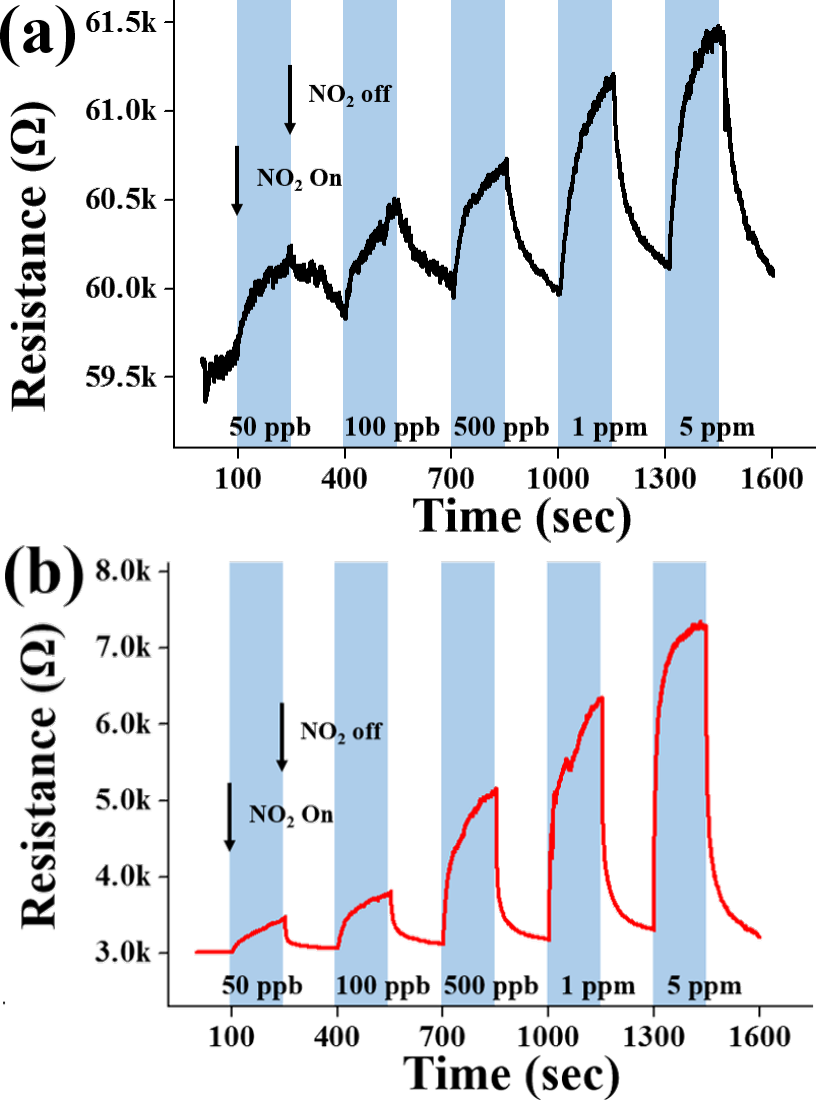
Electrical responses of the gas sensors fabricated from ZnSe nanowires to 50 ppb, 100 ppb, 500 ppb, 1 ppm and 5 ppm NO_2_ gas at room temperature (a) in the dark and (b) under UV (365 nm) illumination at 1.2 mW/cm^2^.

Figure 3a and Figure 3b show the dynamic response of the ZnSe nanowires towards NO_2_ gas at room temperature in the dark and under UV illumination, respectively. The maximum resistance was reached immediately upon exposure to NO_2_ and recovered completely to the initial value after the removal of NO_2_. The resistance increased with increasing NO_2_ concentration. The resistance showed good reversibility during the introduction and exhaust cycles of NO_2_. The ZnSe nanowires showed responses to 50 ppb–5 ppm NO_2_ ranging from ≈101% to ≈102% and from ≈113% to ≈234% in the dark and under UV (365 nm) illumination, respectively. In other words, UV (365 nm) irradiation increased the response of the ZnSe nanowires to 50 ppb–5 ppm NO_2_ by 1.1–2.3 times.

**Figure 4:**
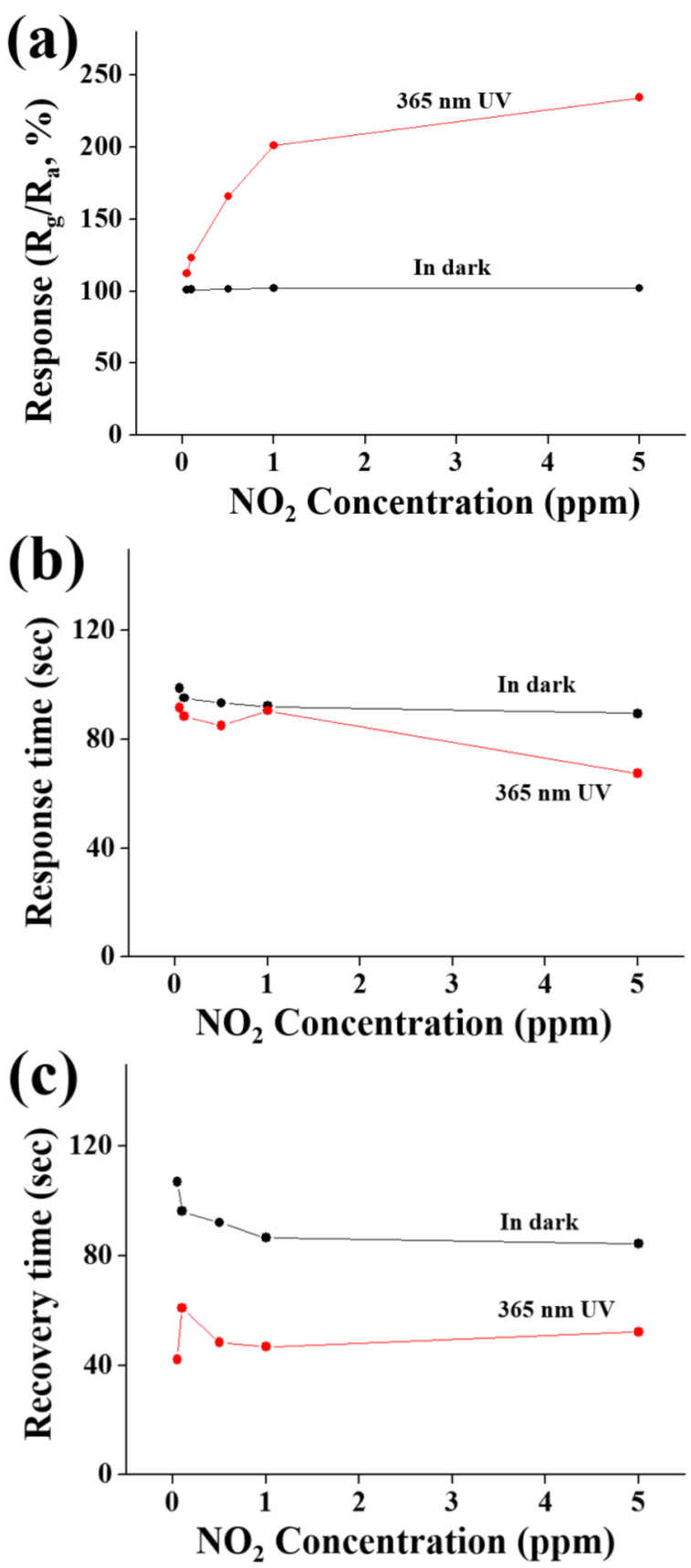
(a) Response, (b) response times and (c) recovery times of the multiple-networked ZnSe nanowire gas sensor to NO_2_ gas in the dark and under 365 nm UV illumination at 1.2 mW/cm^2^.

**Figure 5:**
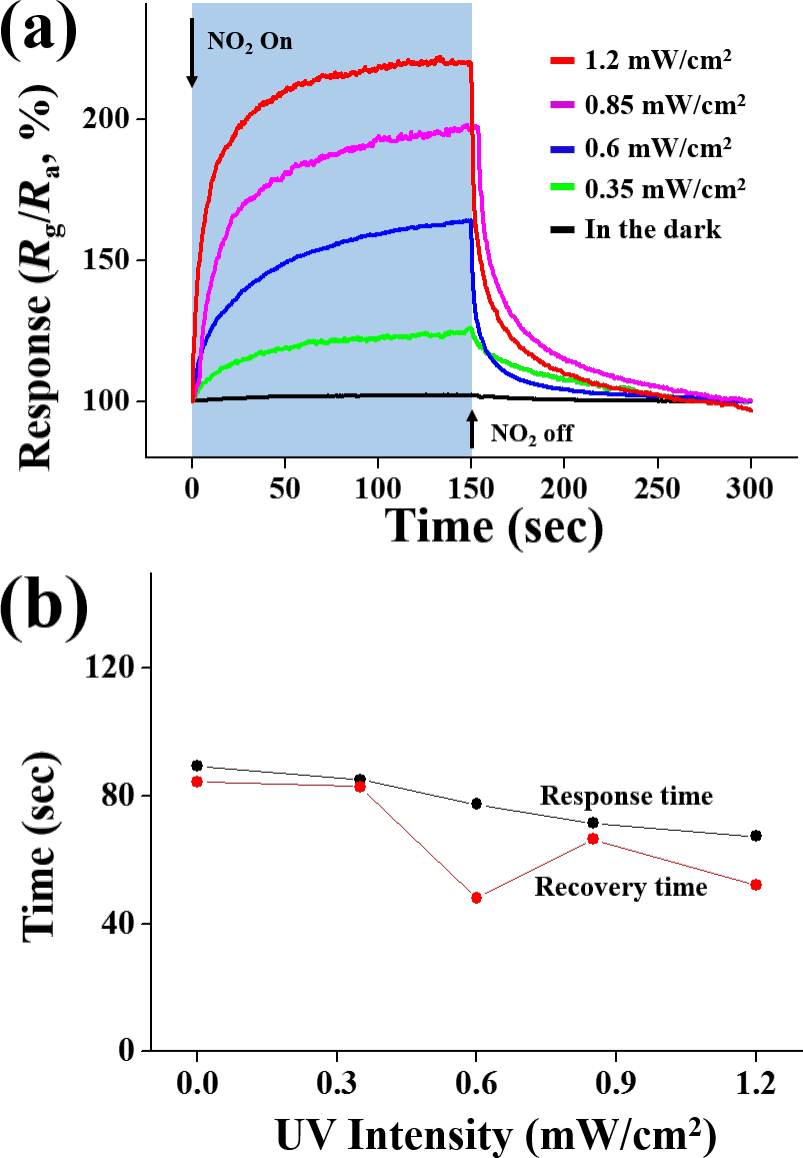
(a) Electrical response and (b) response and recovery times of ZnSe nanowire gas sensors under UV (365 nm) illumination for different UV illumination intensities.

Figure 4a–c show the responses, response times and recovery times of the ZnSe nanowires towards NO_2_ gas at room temperature in the dark and under UV illumination, respectively. This information was determined from that of Figure 3a and Figure 3b. The ZnSe nanowires showed a sufficiently strong response to NO_2_ gas, even at 50 ppb. The response of the ZnSe nanowires to NO_2_ gas tended to increase more rapidly with increasing NO_2_ concentrations under UV illumination than in the dark. Regarding the sensing time, both the response times and recovery times were shorter under UV illumination than in the dark. In particular, recovery times were more than 35 s shorter under UV illumination than in the dark at a NO_2_ gas concentration range from 50 ppb to 5 ppm. Figure 4a–c show the dependence of the response, response time and recovery times of the ZnSe nanowires to 5 ppm NO_2_ gas at room temperature on the illumination intensity of UV light used to illuminate the gas sensors. The response of the nanowires was 102% in the dark. The responses of the nanowires increased from ≈102 to ≈234% with increasing UV illumination intensity from 0 to 1.2 mW/cm^2^ (Figure 4a). Figure 5a shows a strong dependence of the electrical response of the ZnSe nanowires on the UV illumination intensity towards 5 ppm NO_2_ gas at room temperature. The response increased rapidly with increasing UV illumination intensity. On the other hand, Figure 5a and Figure 5b show that both the response time and recovery time of the ZnSe nanowires at room temperature towards 5 ppm NO_2_ gas tend to decrease with the UV illumination intensity. These high responses at room temperature highlight the strong influence of UV irradiation on the response of the nanosensor to NO_2_ gas.

Table 1 compares the responses of the ZnSe nanowires towards NO_2_ synthesized in this study with those of metal oxide semiconductor, 1D nanostructures reported in the literature. The response of the ZnSe nanowires to NO_2_ gas with a lower concentration obtained at room temperature in the dark in this study was stronger than or comparable to those of typical metal oxide, 1D nanostructures, such as ZnO, SnO_2_, In_2_O_3_, and MoO_3_ at higher temperatures and higher NO_2_ concentrations [25–30]. This suggests that the ZnSe nanowires are also a promising candidate as a NO_2_ gas sensor material.

**Table 1:** Responses of various nanomaterial gas sensors to NO_2_ gas.

Nanomaterial	Temperature (°C)	NO_2_ Conc. (ppm)	Response (%)	Ref.

ZnSe NWs (dark)	25	0.05	101	Present work
ZnSe NWs (UV)	25	0.05	113	Present work
ZnO nanorods	300	0.1	35	[24]
ZnO fibers	100	0.4	50	[25]
SnO_2_ NWs	300	10	1.01	[26]
SnO_2_ nanobelts	300	10	1.9	[27]
In_2_O_3_ NWs (multi-NW)	200	0.5	2.1	[28]
In_2_O_3_ NWs (single-NW)	200	0.5	2.6	[28]
MoO_3_ lamellae	225	10	118	[29]

## Conclusion

ZnSe nanowires exhibited responses towards 50 ppb–5 ppm NO_2_ ranging from ≈101% to ≈102% and from ≈113% to ≈234% at room temperature in the dark and under UV (365 nm) illumination, respectively. These responses of ZnSe nanowires were stronger than or comparable to those of typical metal oxide semiconductors reported in the literature, such as ZnO, SnO_2_, In_2_O_3_, and MoO_3_, at higher temperatures and higher NO_2_ concentrations. The ZnSe nanowire sensors cannot be used at high temperatures, such as 300 °C, because of the oxidation of ZnSe, but their sensing performance could be enhanced when used at room temperature under UV illumination. The response of the ZnSe nanowires increased from 0 to ≈234% with increasing UV illumination intensity from 0 to 1.2 mW/cm^2^ and the response time and recovery time of the ZnSe nanowires tended to decrease with increasing UV illumination intensity from 0 to 1.2 mW/cm^2^. The results show that ZnSe nanowires are also a promising nanomaterial for the fabrication of NO_2_ gas sensors when used at room temperature. In addition, the enhanced response of the ZnSe nanowires under UV illumination to NO_2_ gas might be due to (1) modulation of the depletion layer width and (2) the UV-activated adsorption, and desorption of NO_2_ species.

## Experimental

### Synthesis of ZnSe nanowires

Similar to that previously described [31], ZnSe nanowires were synthesized on 3 nm-thick gold (Au) layer-coated, *c*-plane sapphire (Al_2_O_3_(0001)) substrates by the thermal evaporation of ZnSe powders. A quartz tube was mounted inside a horizontal tube furnace. The quartz tube consisted of two temperature zones: zone A at 850 °C and zone B at 700 °C. An alumina boat loaded with pure ZnSe powder was located in zone A, whereas the Au-coated Al_2_O_3_ substrate was placed in zone B. The nitrogen (N_2_) gas flow rate and chamber pressure were 100 cm^3^/min and 1 Torr, respectively. The synthesis process time was 1 h.

#### Characterization of the structure of the nanowires

The morphology and structure of the collected nanowire samples were examined by scanning electron microscopy (SEM, Hitachi S-4200) and transmission electron microscopy (TEM, Philips CM-200), respectively. The crystallographic structures of the samples were determined by glancing angle X-ray diffraction (XRD, Philips X’pert MRD diffractometer) using Cu Kα radiation (λ = 0.15406 nm) at a scan rate of 4°/min, and a 0.5° glancing angle with a rotating detector.

#### Preparation of sensors and gas sensing tests

ZnSe nanowire samples were dispersed ultrasonically in a mixture of deionized water (5 mL) and isopropyl alcohol (5 mL), and dried at 90 °C for 30 min. A slurry droplet containing the nanowires (10 µL) was placed onto the SiO_2_-coated Si substrates equipped with a pair of interdigitated (IDE) Ni (≈200 nm)/Au (≈50 nm) electrodes with a gap of 20 μm. The flow-through technique was used to test the gas sensing properties. All measurements were performed in a temperature-stabilized, sealed chamber with a constant flow rate of 200 cm^3^/min at 25 °C under 50% RH. The NO_2_ concentration was controlled by mixing NO_2_ gas with synthetic air at different ratios. The detailed procedures for the sensor fabrication and sensing test are reported elsewhere [32]. The electrical resistance of the gas sensors was determined in the dark and under UV light (λ = 365 nm) illumination at intensities ranging from 0.35 to 1.2 mW/cm^2^ by measuring the electric current between the Ni/Au IDEs at 1 V and at room temperature. The response was defined as (*R*_g_/*R*_a_) × 100% for NO_2_ gas, where *R*_g_ and *R*_a_ are the electrical resistances of the sensors in the target gas and air, respectively.

## References

[1] Park S, An S, Ko H, Lee C (2014). Mater Chem Phys.

[2] Ma R, Bando Y (2003). Chem Phys Lett.

[3] Mirov S B, Fedorov V V, Graham K, Moskalev I S, Badikov V V, Panyutin V (2002). Opt Lett.

[4] Katayama K, Matsubara H, Nakanishi F, Nakamura T, Doi H, Saegusa A, Mitsui T, Matsuoka T, Irikusa M, Takebe T (2000). J Cryst Growth.

[5] Okuyama H (2000). Trans Inst Electron, Inf Commun Eng, Sect E.

[6] Chang S J, Su Y K, Chen W R, Chen J F, Lan W H, Lin W J, Cherng Y T, Liu C H, Liaw U H (2002). IEEE Photonics Technol Lett.

[7] (2014). Dow Chemical Corporate Website - The Dow Chemical Company.

[8] Kim H, Jin C, An S, Lee C (2012). Ceram Int.

[9] Kolmakov A, Zhang Y, Cheng G, Moskovits M (2003). Adv Mater.

[10] Liu Y, Koep E, Liu M (2005). Chem Mater.

[11] Law M, Kind H, Messer B, Kim F, Yang P (2002). Angew Chem.

[12] Lin Y-H, Huang M-W, Liu C-K, Chen J-R, Wu J-M, Shih H-C (2009). J Electrochem Soc.

[13] Kim H S, Jin C H, Park S H, Kim S I, Lee C (2012). Sens Actuators, B.

[14] Ramgir N S, Mulla I S, Vijayamohanan K P (2005). Sens Actuators, B.

[15] Wan Q, Wang T H (2005). Chem Commun.

[16] Kolmakov A, Klenov D O, Lilach Y, Stemmer S, Moskovits M (2005). Nano Lett.

[17] Kuang Q, Lao C-S, Li Z, Liu Y-Z, Xie Z-X, Zheng L-S, Wang Z L (2008). J Phys Chem C.

[18] Wright J S, Lim W, Gila B P, Pearton S J, Johnson J L, Ural A, Ren F (2009). Sens Actuators, B.

[19] Tamaki J, Shimanoe K, Yamada Y, Yamamoto Y, Miura N, Yamazoe N (1998). Sens Actuators, B.

[20] Park S, Ko H, Kim S, Lee C (2014). ACS Appl Mater Interfaces.

[21] Jin C, Park S, Kim H, Lee C (2012). Sens Actuators, B.

[22] Comini E, Cristalli A, Faglia G, Sberveglieri G (2000). Sens Actuators, B.

[23] Gong J, Li Y, Chai X, Hu Z, Deng Y (2010). J Phys Chem C.

[24] Lu G, Xu J, Sun J, Yu Y, Zhang Y, Liu F (2012). Sens Actuators, B.

[25] Park S H, An S Y, Ko H S, Jin C H, Lee C (2012). ACS Appl Mater Interfaces.

[26] Baratto C, Sberveglieri G, Onischuk A, Caruso B, di Stasio S (2004). Sens Actuators, B.

[27] Kim H, An S, Jin C, Lee C (2012). Curr Appl Phys.

[28] Law M, Kind H, Messer B, Kim F, Yang P (2002). Angew Chem, Int Ed.

[29] Moon S E, Kim E-K, Lee H-Y, Lee J-W, Park J, Park S-J, Kwak J-H, Park K-H, Kim J, Jo G-H (2009). J Korean Phys Soc.

[30] Rahmani M B, Keshmiri S H, Yu J, Sadek A Z, Al-Mashat L, Moafi A, Latham K, Li Y X, Wlodarski W, Kalantar-zadeh K (2010). Sens Actuators, B.

[31] Lee C, Jin C, Kim H, Kim H W (2010). Curr Appl Phys.

[32] Oh E, Choi H-Y, Jung S-H, Cho S, Kim J C, Lee K-H, Kang S-W, Kim J, Yun J-Y, Jeong S-H (2009). Sens Actuators, B.

